# Cerebral Perfusion Pressure Insults and Associations with Outcome in Adult Traumatic Brain Injury

**DOI:** 10.1089/neu.2016.4807

**Published:** 2017-08-15

**Authors:** Fabian Güiza, Geert Meyfroidt, Ian Piper, Giuseppe Citerio, Iain Chambers, Per Enblad, Pelle Nillson, Bart Feyen, Philippe Jorens, Andrew Maas, Martin U. Schuhmann, Rob Donald, Laura Moss, Greet Van den Berghe, Bart Depreitere

**Affiliations:** ^1^ Department of Intensive Care Medicine, University Hospitals Leuven, Leuven, Belgium.; ^2^ Department of Clinical Physics, Southern General Hospital, Glasgow, United Kingdom.; ^3^ Department of Perioperative Medicine and Intensive Care Medicine, San Gerardo Hospital, Monza, Italy.; ^4^ Medical Physics, James Cook University Hospital, Middlesborough, United Kingdom.; ^5^ Department of Neurosciences, Neurosurgery, Uppsala, Sweden.; ^6^ Department of Neurosurgery, Antwerp University Hospital, Edegem, Belgium.; ^7^ Department of Intensive Care Medicine, Antwerp University Hospital, Edegem, Belgium.; ^8^ Klinik für Neurochirurgie, Universitätsklinikum Tübingen, Tübingen, Germany.; ^9^ School of Mathematics and Statistics, University of Glasgow, Glasgow, United Kingdom.; ^10^ Department of Clinical Physics and Bioengineering, NHS Greater Glasgow & Clyde, Glasgow, United Kingdom.; ^11^ Department of Neurosurgery, University Hospitals Leuven, Leuven, Belgium.

**Keywords:** adults, cerebral perfusion pressure, cerebrovascular autoregulation, traumatic brain injury

## Abstract

The definition of cerebral perfusion pressure (CPP) secondary insults in severe traumatic brain injury remains unclear. The purpose of the present study is to visualize the association of intensity and duration of episodes below or above CPP thresholds and outcome. The analysis was based on prospectively collected minute-by-minute intracranial pressure (ICP) and blood pressure data and outcome from 259 adult patients. The relationship of episodes of CPP below or above a certain threshold for certain duration with the 6-month Glasgow Outcome Score was visualized separately for episodes of active or deficient autoregulation (AR). In adults ≤65 years, an almost exponential transition curve separates the episodes of CPP associated with better outcomes from the episodes of low CPP associated with worse outcomes, indicating that lower CPP could only be tolerated for a brief time. Analysis of episodes of high CPP again showed a time-intensity dependent association with outcome. When combining the two plots, a safe CPP zone between 60 and 70 mm Hg could be delineated—however, only for AR active insults. The AR status predominantly affected the transition curve for insults of low CPP. Episodes with ICP >25 mm Hg were associated with poor outcome regardless of CPP. In the present study, the CPP pressure-time burden associated with poor outcome was visualized. A safe zone between 60 and 70 mm Hg could be identified for adults ≤65 years, provided AR was active and ICP was ≤25 mm Hg. Deficient AR reduces the tolerability for low CPP.

## Introduction

Traumatic brain injury (TBI) is a significant health problem worldwide. In Europe, the overall incidence of fatal and hospitalized TBI is 262/100,000/year.^1^ An estimated 43.3% of hospitalized patients with TBI in the United States experience long-term disability.^2^ The management of severe TBI focuses on avoiding additional ischemic damage from preventable or reversible secondary insults. Intracranial hypertension has long been identified as an insult associated with poorer outcomes.^3–5^ Therefore, it is recommended that intracranial pressure (ICP) be monitored in salvageable patients with severe TBI and to treat patients with ICP above 22 mm Hg.^6^ It is evident that “22” is not a magical number, and it was recently demonstrated by our group that the relation between ICP level and outcome is not only time dependent, but that ICP tolerability also depends on age and pressure autoregulation (AR) capacity.^7^

While the relation between ICP and outcome is still rather straightforward, however, guidelines on blood pressure management in severe TBI seem more difficult to produce. Cerebral perfusion pressure (CPP) is the driving force of cerebral blood flow, expressed as the difference between the mean arterial blood pressure (MAP) and ICP. Within a certain range of CPP, the cerebral vasculature has the ability to maintain constant cerebral blood flow, which is—among others—based on alterations in basal tone and subsequent diameter changes of pial arterioles.^8^ This capacity for “pressure AR” can be deficient after TBI.^9^ Moreover, with intact AR, low CPP can induce a vasodilatory cascade resulting in raised ICP.

The latter phenomenon led Rosner and associates^10^ to formulate the recommendation that CPP in severe TBI situations should be kept above 70 mm Hg. This concept, however, could not be demonstrated to produce better outcomes in a randomized controlled trial.^11^ At the same time the Lund concept, which emphasized avoidance of the use of vasopressors and accepted CPP down to 50 mm Hg for adults, did not result in poorer outcomes.^12^ An explanation for this apparent paradox was sought mainly in the dynamic, and hence more complex, behavior of pressure AR capacity or deficiency after TBI.^13,14^

Real-time monitoring of cerebrovascular pressure reactivity through parameters such as the Pressure Reactivity Index (PRx)^14^ or Low frequency Autoregulation Index (LAx)^15^ has enabled the identification of CPP ranges in which AR is more active,^15–17^ and in retrospective series, higher percentages of time of actual CPP contained within these zones were associated with better outcomes.^15,18^ At present, pressure reactivity monitoring is predominantly performed in centers with a research interest in severe TBI.

In the latest version of the Brain Trauma Foundation guidelines, the chapter on CPP thresholds is limited to a level IIB recommendation that the CPP value to target lies within the range of 60 to 70 mm Hg.^6^ For a parameter subject to variability such as CPP, however, it may be helpful to the clinician to have more insight into the burden on outcome of CPP insults in terms of severity and duration.

The aim of the present study is to assess the effect of CPP insults, according to varying definitions of intensity and duration, on functional outcome at six months based on prospective data from continuously monitored adult patients with severe TBI. In addition, the impact of ICP, cerebrovascular AR, age, and decompressive craniectomy (DC) on the capacity to tolerate the CPP insults is investigated.

## Methods

### Patients and data

The study population consisted of 259 patients with severe TBI aged 16 years and older:

• There were 164 patients included from the Brain-IT database,^19^ a multicenter data collection between March 2003 and July 2005 (Newcastle, Uppsala, Monza, Leuven, Heidelberg, Iasi, Barcelona, Leipzig, Kaunas, Turin, Novara, Vilnius, Milan, Glasgow, Mannheim, Edinburgh, Southampton, Cambridge, Zurich, London, Göteborg). The Multi-Centre Research Ethics Committee for Scotland MREC/02/0/9 granted the use of these data for scientific purposes on February 14, 2002.
• The data of the remaining 95 adult patients were collected from four centers: 38 from the San Gerardo Hospital in Monza, Italy, between March 2010 and April 2013; 25 from the University Hospitals, Leuven, Belgium, between September 2010 and September 2013; 20 from the University Hospital, Antwerp, Belgium, between May 2010 and June 2013; and 12 from the University Hospital, Tübingen, Germany, between February and December 2009. Local Ethical Committee approval to use the anonymized data for this analysis was obtained in all centers.

Data collection included baseline risk factors (age, sex, admission Glasgow Coma Score (GCS), admission pupil reactivity), and minute-by-minute ICP and MAP monitoring data. Monitoring data were recorded in minute-by-minute resolution through specifically designed “BrainIT data collection software” for the BrainIT study.^19^ For the later cohort, data were recorded through the centers' Patient Data Management System, in minute-by-minute resolution in Leuven, Tübingen, and Monza, and in second-by-second resolution in Antwerp. For the Antwerp patients, the median value per minute was used in this analysis. Minute-by-minute signals from all data sets were reviewed independently by two senior clinicians in Leuven (GM, BD), and obvious artifacts at visual inspection of data traces were removed.

Finally, a correction of the CPP values for arterial blood pressure transducer height was made based on the information that was obtained on the height of the arterial blood pressure transducer and of the head of the bed. For patients in whom the transducer was at cardiac atrium level and who received nursing care with the head of the bed elevated at 30 degrees, 10 mm Hg was subtracted from the registered CPP. This was the case in 101/259 patients (39.0%).

To investigate the effect of DC and old age on the capacity to tolerate CPP insults, the analyses were performed separately for three groups: patients ≤65 years who did not undergo DC (*n* = 179), patients >65 years who did not undergo DC (*n* = 35), and patients ≤65 years who underwent DC (*n* = 37). The details of the three groups are presented in Table 1. The group of patients ≤65 years who did not undergo DC was considered the standard group on which the analyses assessing the effect of AR and ICP were performed (see below).

**Table 1.** Patient Demographic, Injury, and Outcome Data

	*Cohort ≤65 years of age without DC*	*Cohort >65 years of age without DC*	*Cohort ≤65 years of age with DC*
Number of patients (*n*)	179	35	37
LOS days, median (IQR)	14 (7–23)	14 (6.5–24.5)	24.5 (14–31)
Age, median (IQR)	36 (24–50.1)	72 (69–75.5)	36.9 (23–52.3)
Sex (% male)	82.1	71.4	83.8
Pupil reactivity			
None (%)	9.5	20.0	18.9
One (%)	12.3	5.7	10.8
Two (%)	72.1	68.6	67.6
Unknown, untestable or missing (%)	6.1	5.7	2.7
GCS total, median (IQR)	7 (4–10)	7.5 (4.5–11)	7 (3–11)
Unknown, untestable or missing (%)	5.6	8.6	10.8
GCS motor, median (IQR)	4 (1–5)	5 (2–5)	4 (1–5)
Unknown, untestable or missing (%)	2.8	2.7	10.8
CPP monitored time (days)	5.8 (2.9–10.7)	5.1 (2.7–9)	10.7 (5.4–12.2)
Percent valid CPP monitored time^*^	94.9 (89.6–97.6)	95.0 (83.1–97.6)	93.0 (81.1–96.7)
GOS at six months, median (IQR)	4 (3–5)	3 (1–4.8)	4 (3–5)
GOS 1 = death (*n*; %)	23; 12.9	13; 37.1	5; 13.5
GOS 2 = vegetative (*n*; %)	4; 2.2	3; 8.6	2; 5.4
GOS 3 = severe disability (*n*; %)	49; 27.4	9; 25.7	11; 29.7
GOS 4 = moderate disability (*n*; %)	43; 24.0	1; 2.9	4; 10.8
GOS 5 = low disability (*n*; %)	60; 33.5	9; 25.7	15; 40.5

^*^ Non-valid data were because of monitor disconnections, data loss during patient transport. or artefacts removed manually.

DC, decompressive craniectomy; LOS, length of stay; IQR, interquartile range; GCS, Glasgow Coma Scale; CPP, cerebral perfusion pressure; GOS, Glasgow Outcome Scale.

### Outcome

Functional outcome was assessed at 6 months in all centers using the Glasgow Outcome Score (GOS).^20^

### Visualization method

The method developed in our group for visualizing the univariate association between insult and outcome and that was used for assessing the pressure and time burden of intracranial hypertension in Güiza and colleagues^7^ was applied in the current analysis investigating the relationship between CPP and outcome. For CPP, two types of insults are defined: insults characterized by CPP dropping below a certain pressure value for a certain duration of time as well as insults characterized by CPP exceeding a certain pressure value for a certain duration of time. Hence, the visualization was performed separately for insults of low CPP and insults of high CPP. The Pearson correlation coefficient between the number of insults of a certain intensity and duration and GOS was expressed by a graded color code: negative correlations in red (−1 in dark red) and positive correlations in blue (+1 in dark blue). The contour for zero correlation was highlighted in black and defined as the “transition curve.” For more details about the visualization method, we refer to Güiza and coworkers.^7^ Analytic routines were programmed in Matlab^®^ (MathWorks Inc, Natick, MA).

### Role of cerebrovascular pressure reactivity expressed by LAx

The LAx, expressing cerebrovascular pressure reactivity as an indicator of pressure AR, is calculated as the moving median of the correlation coefficients between ICP and MAP for the past time intervals of 3, 5, 10, 20, 30, 60, and 90 min, using minute-by-minute resolution data.^15^ The LAx was calculated at every minute during the monitoring period and average LAx for every CPP insult period, which allowed for discriminating CPP insults associated with “active” AR (if average LAx <0) or “passive” AR (if average LAx ≥0). Hence, the relationship between CPP insults and outcome was visualized separately considering active or passive episodes exclusively.

### Role of ICP

The relationship between CPP insults and outcome was visualized separately for CPP insults associated with average ICP ≤25 mm Hg and for CPP insults associated with average ICP >25 mm Hg to examine the relative effect of ICP and CPP insults on outcome. The value of 25 mm Hg was chosen as the discriminator because ICP above 25 mm Hg was associated with poor outcome independent of the insult duration in our ICP article.^7^

### Multivariate analysis

Multivariate logistic regression models for death were calculated to investigate the independent association of the cumulative dose of CPP insult burden with outcome. Therefore, the percentage of total monitoring time each patient had CPP insults associated with worse GOS (i.e., time spent in the red zone) was used as an independent variable, together with the IMPACT model core variables age, pupils, and admission Glasgow motor score.^21^ The percentage of monitoring time spent in the red zone was based on a new calculation, in which for each patient, for every minute of monitoring it was checked whether this minute fulfilled criteria of belonging to one or more of the several episodes negatively associated with outcome (i.e., red zone). The time in the red zone was then divided by the total monitoring time of that patient.

### Impact of CPP policy

The database contains patients treated before and after the publication of the third revision of the Brain Trauma Foundation guidelines in 2007, in which the recommendation to aim at CPP higher than 70 mm Hg was abandoned.^22^ To assess whether CPP policy confounded the current analysis, the median of the patients' median CPP was calculated per center. For the two centers that contributed data to the cohort before and after 2007, the median was calculated separately for the patients of both periods. Next, the visualization method was applied separately for centers with a median CPP below and above 70 mm Hg to assess its validity independent of CPP policy.

## Results

**FIG. 1.**
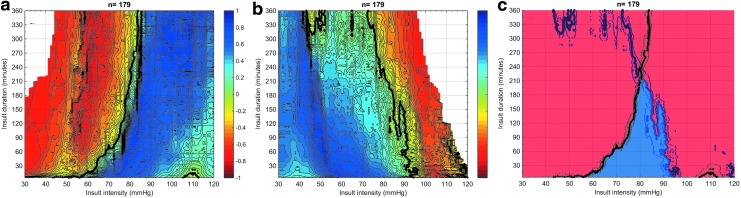
Visualization of correlation between the Glasgow Outcome Score (GOS) and average number of cerebral perfusion pressure (CPP) insults per GOS category for adults ≤65 years without decompressive craniectomy, *n* = 179. Each dot in the graph represents a CPP insult—i.e., an episode of CPP defined by an intensity threshold (X-axis) and a certain duration (Y-axis). The univariate correlation between the average number of a certain CPP insult defined by severity (X-axis) and duration (Y-axis) and each GOS category is color-coded with blue representing a positive correlation and red representing a negative correlation. The contour of zero correlation is highlighted in black. 1a: insults of low CPP (i.e., CPP on the X-axis expresses the threshold defining insults where CPP went below this threshold for a certain duration). 1b: insults of high CPP (i.e., CPP on the X-axis expresses the threshold defining insults where CPP went above this threshold for a certain duration). 1c: low CPP (black) and high CPP (blue) transition curves plotted together.

For adults ≤65 years who did not undergo DC (*n* = 179), the visualization of the association between GOS and the intensity and duration of CPP insults is shown in Figure 1. In both instances (Fig. 1a and Fig. 1b), a blue zone (representing episodes of CPP associated with higher GOS) and red zone (representing episodes of CPP associated with lower GOS) can be observed, separated by a sharply demarcated transition curve. Figure 1a shows that insults below a certain threshold were associated with worse outcomes and that the lower the CPP value, the shorter the duration they could be tolerated.

In the present analysis, for instance, transition to worse outcomes below CPP of 60 mm Hg occurred at a duration of 10 min, while CPP of 50 mm Hg could not be tolerated at all. Figure 1b shows a similar parabolic course of the association for insults above a certain CPP threshold with transition to worse outcomes occurring at shorter durations for the higher CPP values. When combining both plots (Fig. 1c), it becomes apparent that no universal CPP value is associated with good outcome for longer than 210 min. In other words, a common and static safe CPP zone could not be found. The graphs are cut off at 360 min; they became blurred at longer durations because of the numbers of insults of such long durations steeply falling down.

**FIG. 2.**
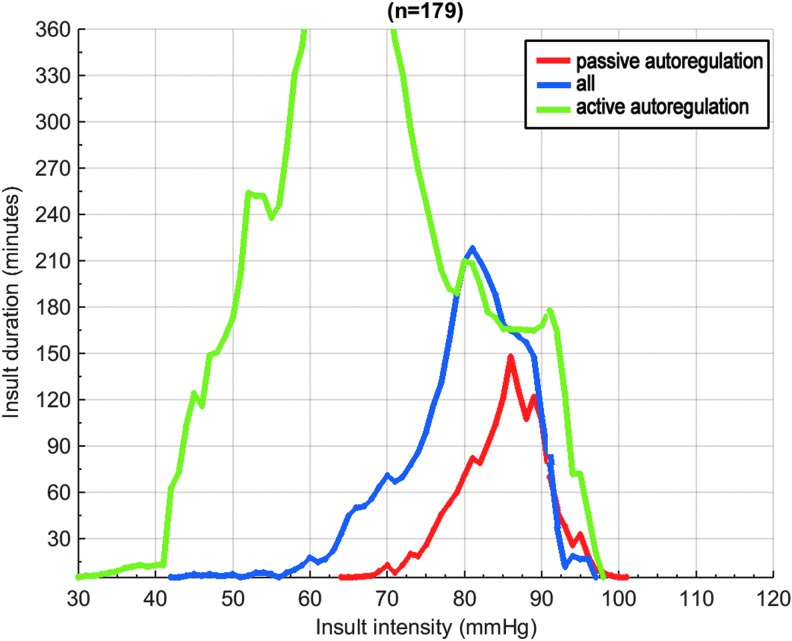
Comparison of cerebral perfusion pressure insult transition curves for episodes with active autoregulation (LAx <0; green) for episodes with passive autoregulation (LAx ≥0; red) and for all episodes (blue). Transition curves are the lines with 0 correlation between CPP insults and outcome. Analysis performed in adults ≤65 years without decompressive craniectomy, *n* = 179.

Figure 2 shows the transition curves for both insults of low CPP and insults of high CPP in adults ≤65 years without DC, separately for insults during which AR was active and insults during which AR was passive (the corresponding color-coded plots are available in Supplementary Fig. 1; see online supplementary material at ftp.liebertpub.com). In active episodes, there was a pronounced shift to the left of the transition curve for low CPP, while the transition curve for high CPP slightly shifted to the right, resulting in a zone between 60 and 70 mm Hg in which CPP was associated with better outcome up to 360 min. In passive episodes, the transition curve for high CPP remained fairly constant, but the transition curve for low CPP showed a pronounced shift to the right, resulting in the finding that no CPP value was associated with good outcome for longer than 90 min.

**FIG. 3.**
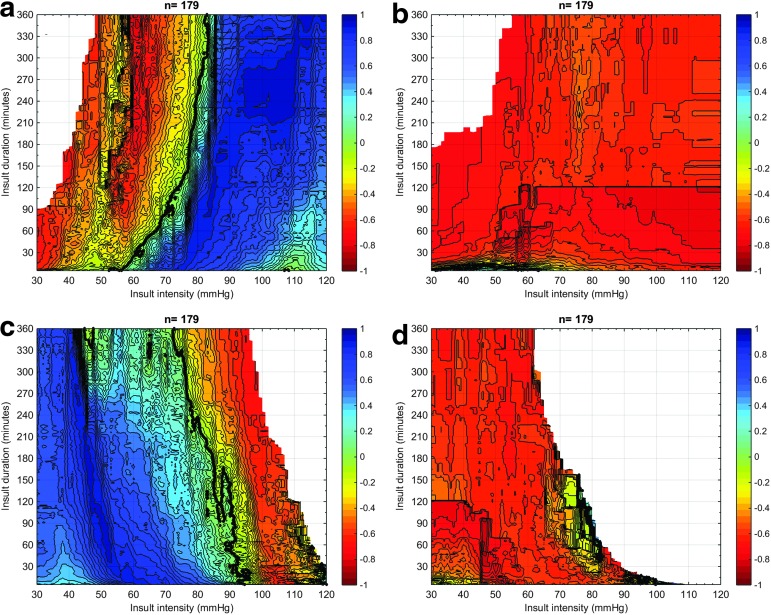
Visualization of correlation between Glasgow Outcome Scale (GOS) and average number of cerebral perfusion pressure (CPP) insults according to an intracranial pressure (ICP) threshold of 25 mm Hg. Analysis performed in adults ≤65 years without decompressive craniectomy, *n* = 179. The univariate correlation between the average number of a certain CPP insult defined by severity (X-axis) and duration (Y-axis) and each GOS category is color-coded with blue representing a positive correlation and red representing a negative correlation. The contour of zero correlation is highlighted in black. 3a: insults of low CPP for ICP ≤25 mm Hg; 3b: insults of low CPP for ICP >25 mm Hg; 3c: insults of high CPP for ICP ≤25 mm Hg; 3d: insults of high CPP for ICP >25 mm Hg.

In Figure 3, the visualization of the relation between GOS and CPP insults for adults ≤65 years without DC is repeated, however, while only selecting episodes where ICP was ≤25 mm Hg (Fig. 3a and 3c) or only selecting episodes where ICP was >25 mm Hg (Fig. 3b and 3d). The graphs for ICP ≤25 mm Hg are almost identical to the graphs depicting all insults, while the graphs for ICP >25 mm Hg are overwhelmingly red: i.e., all insults are associated with poor outcomes.

As for elderly persons >65 years without DC (*n* = 35), no transition curves could be discerned, outcomes being overall poor (see Supplementary Fig. 2; see online supplementary material at ftp.liebertpub.com). In adults ≤65 years who underwent a DC (*n* = 37), no meaningful transition curves could be identified either (see Supplementary Fig. 3; see online supplementary material at ftp.liebertpub.com). The percentage of monitoring time with active AR was 44.5% for the adults ≤65 years without DC and 38.2% for the elderly without DC.

**FIG. 4.**
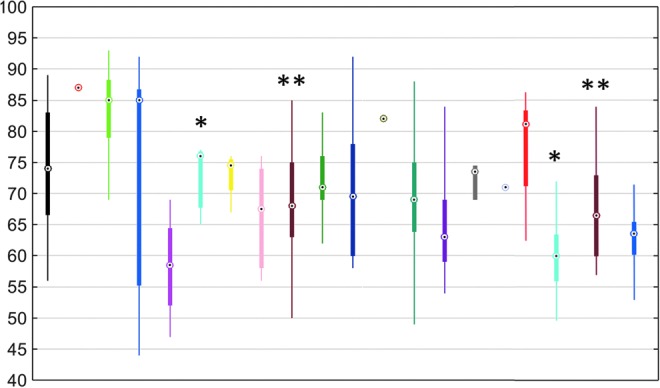
Per center distribution (median, interquartile range, and total range) of patients' median cerebral perfusion pressure throughout the intensive care unit stay. Each color represents a center. * and ** indicate centers that are depicted twice: once for the cohort 2003–2005 and once for the cohort 2009–2013.

Nine centers had a median of patients' median CPP below 70 mm Hg and 11 above 70 mm Hg (Fig. 4). When the color-coded plots visualizing the burden of CPP insults for adults ≤65 years without DC are depicted separately for both groups of centers, the transition curve patterns remain similar (see Supplementary Fig. 4; see online supplementary material at ftp.liebertpub.com).

In the multivariate analysis, the percentage of monitoring time each adult spent in the red zone (based on the transition curves of the active adults ≤65 years without DC) was an independent predictor of mortality (odds ratio [OR] 4.79 [1.02–22.46]; *p* = 0.04, Supplementary Table 1; see online supplementary material at ftp.liebertpub.com).

## Discussion

We used the method for visualizing the burden of intracranial hypertension in patients with severe TBI developed by our group^7^ to visualize the burden of CPP insults in terms of poor outcomes associated with episodes in which certain CPP thresholds are exceeded for certain durations. In the group of adults ≤65 years who did not undergo DC, we found that positive and negative associations between the occurrence of insults and outcome were separated by a well demarcated transition curve, which had an exponential course similar to that seen in the ICP study.^7^ The lower the CPP, the shorter the time this could be tolerated, and the same was true for high CPP. Hence, both transition curves for CPP have to be considered together, which results in a safe zone in between the two curves. Only in active AR, however, the curves spread apart sufficiently to result in a CPP zone that was associated with good outcomes for a longer period. In deficient AR, the curves came closer, and no CPP value was safe for more than 90 min.

Although this finding may have been influenced by unknown confounders, the observation is congruent with the phenomenon of AR as a stable cerebral blood flow (CBF) plateau regardless of MAP in the Lassen representation.^8^ Strikingly, AR status predominantly affected the transition curve for insults of low CPP. In the older age group and DC group, the sample size was probably too low to draw conclusions. Still, however, it was striking that the graph pattern observed in the younger adults without decompression did not emerge here. When analyzing the total patient cohort including elderly and DC patients, relatively similar graphs as in Figure 1 were produced, but with a wider area of transition between good and poor outcome instead of sharper transition curves.

The Lassen representation of the cerebrovascular AR as a CBF plateau for MAP between 60 and 150 mm Hg^8^ has been criticized as an oversimplification and more importantly because it was based on a disputable methodology of grouping populations from different studies.^23,24^ From physiological research, AR emerges as a complex feedback mechanism with overlapping and interacting pathways, including intrinsic vasomotor mechanisms, peripheral autonomic vasomotor regulations, and central intrinsic neural pathways. The actual intra-individual range of effective AR is thought to be as narrow as approximately 10 mm Hg.^25^ Also, AR would seem to be better at buffering increases versus decreases of MAP,^26,27^ which is inverse to our observation that AR had the most pronounced effect on the “tolerance curve” for low CPP. Noteworthy, the effect of AR on the transition curve for low CPP in the present analysis concerns a wider pressure range than the effect of AR on the ICP transition curve in Güiza and associates.^7^

In several studies, it has been reported that the impairment of AR in TBI is a dynamic phenomenon.^14,28,29^ In active AR, a safe CPP zone between 60 and 70 mm Hg for up to 6 h is depicted in the current study. How this safe zone looks like for longer periods cannot be concluded from the present study. Variability of blood pressure and ICP in real life, as in the present study, however, may question the relevance of this issue. The safe zone is congruent with the AR stable CBF plateau concept, but a dynamic component is added—i.e., the actual range of safe CPP is wider than 60 to 70 mm Hg, but outside the 60 to 70 mm Hg, it is time dependent.

This is not in conflict with the literature on optimal CPP.^16–18^ First, there is a different perspective. The optimal CPP method searches for CPP values associated with active AR (based on PRx or LAx) and then finds a relationship with outcome. The current study investigated the relationship between CPP and outcome and then looked at the influence of AR (using LAx). Second, associations with favorable outcome can be found far outside the 60 to 70 mm Hg range, but then they are time dependent. The optimal CPP method, within the scope of the CPP range the patient has been in, will pick up time-dependent CPP zones associated with active AR in this wider range. Future studies, both clinical studies as well as validation studies of clinical tools in animal AR models, will be required to refine our understanding of AR and optimal CPP. What can be concluded from the current article and our ICP article^7^ is that active AR improves the tolerability for insults of high ICP and low CPP. Consequently, it is likely beneficial to bring the patient with TBI in a state of active AR.

Insults of CPP <50 mm Hg were hardly tolerated, and ICP >25 mm Hg was associated with poor outcome regardless of CPP. The latter two findings were also demonstrated in Güiza and coworkers.^7^ That the impact of high ICP on outcome overrules the effect of CPP, however, should not be interpreted as if the management of CPP is unimportant during episodes of high ICP. It may well be that, more than the pressure level itself, high ICP reflects the global seriousness of the patient's condition, and hence is reflected in its profound relationship with poor outcome.

The current study inevitably has limitations. First, the sample size remains relatively small. Second, the data incorporate therapeutic influences, which cannot be removed. As we have shown, however, CPP policies did not affect the CPP/outcome transition curves. Third, although efforts were made to correct for the height at which arterial blood pressure was zeroed, some degree of uncertainty cannot be excluded. When we analyzed the data without correction, however, the influence on the graphs was minimal and the interpretation remained similar. Fourth, longer-staying patients have a proportionally higher representation in the total monitoring time. To rule out bias from this phenomenon, the visualization plots were recalculated using daily averages of the number of insults. The graphs remained unchanged (Supplementary Fig. 5; see online supplementary material at ftp.liebertpub.com). Fifth, artifacts in the monitoring data were manually removed by two clinical experts, and we cannot exclude that some artifacts went unnoticed. Sixth, although the results seemed robust, we cannot exclude an influence from confounders that were not analyzed. Also, it should be kept in mind that this analysis reports on correlative and not necessarily causative relationships. Seventh, AR was considered as active (LAx negative) or passive (LAx positive), while in reality it does not behave as an on-off phenomenon, and the threshold of “0” is debatable. Moreover, it should be taken into account that LAx produces a rough estimation of changes of AR capacity over longer periods, with lower resolution than the actual phenomenon. Finally, LAx may behave noisy when only considered in short time intervals, which should be taken into account when interpreting the influence of AR on the very short CPP insult periods.

The present study attempts to produce a better insight into insults of low and high CPP. The results support that there is an impact of AR on the relationship between CPP and outcome and demonstrate the important influence of the time factor. From this, it is impossible to produce fixed CPP thresholds. This emphasizes the need for improving our understanding of cerebrovascular pathophysiology in TBI and including this knowledge into smart(er) monitoring tools.

## Supplementary Material

Supplemental data

Supplemental data

Supplemental data

Supplemental data

Supplemental data

Supplemental data

## References

[1] Peeters W., van den Brande R., Polinder S., Brazinova A., Steyerberg E.W., Lingsma H.F., and Maas A.I. (2015). Epidemiology of traumatic brain injury in Europe. Acta Neurochir. (Wien) 157, 1683–1696 26269030 10.1007/s00701-015-2512-7 PMC4569652

[2] Selassie A.W., Zaloshnja E., Langlois J.A., Miller T., Jones P., and Steiner C. (2008). Incidence of long-term disability following traumatic brain injury hospitalization, United States. J. Head Trauma Rehabil. 23, 123–131 18362766 10.1097/01.HTR.0000314531.30401.39

[3] Narayan R.K., Kishore P.R., Becker D.P., Ward J.D., Enas G.G., Greenberg R.P., Domingues Da Silva A., Lipper M.H., Choi S.C., Mayhall C.G., Lutz H.A. 3rd, and Young H.F. (1982). Intracranial pressure: to monitor or not to monitor? A review of our experience with severe head injury. J. Neurosurg. 56, 650–659 7069477 10.3171/jns.1982.56.5.0650

[4] Marmarou A., Anderson R.L., Ward J.D., Choi S.C., Young H.F., Eisenberg H.M., Foulkes M.A., Marshall L.F., and Jane J.A. (1991). Impact of ICP instability and hypotension on outcome in patients with severe head trauma. J. Neurosurg. 75, S59–S66

[5] Saul T.G., and Ducker T.B. (1982). Effect of intracranial pressure monitoring and aggressive treatment on mortality in severe head injury. J. Neurosurg. 56, 498–503 6801218 10.3171/jns.1982.56.4.0498

[6] Carney N., Totten A.M., O'Reilly C., Ullman J.S., Hawryluk G.W., Bell M.J., Bratton S.L., Chesnut R., Harris O.A., Kissoon N., Rubiano A.M., Shutter L., Tasker R.C., Vavilala M.S., Wilberger J., Wright D.W., and Ghajar J. (2017) Guidelines for the management of severe traumatic brain injury, Fourth Edition. Neurosurgery 80, 6–15 27654000 10.1227/NEU.0000000000001432

[7] Güiza F., Depreitere B., Piper I., Citerio G., Chambers I., Jones P.A., Lo T.Y., Enblad P., Nillson P., Feyen B., Jorens P., Maas A., Schuhmann M.U., Donald R., Moss L., Van den Berghe G., and Meyfroidt G. (2015) Visualizing the pressure and time burden of intracranial hypertension in adult and paediatric traumatic brain injury. Intensive Care Med. 41, 1067–1076 25894624 10.1007/s00134-015-3806-1

[8] Lassen N.A. (1959). Cerebral blood flow and oxygen consumption in men. Physiol. Rev. 39, 183–238 13645234 10.1152/physrev.1959.39.2.183

[9] Bouma G.J., Muizelaar J.P., Bandoh K., and Marmarou A. (1992). Blood pressure and intracranial pressure-volume dynamics in severe head injury: relationship with cerebral blood flow. J. Neurosurg. 77, 15–19 1607958 10.3171/jns.1992.77.1.0015

[10] Rosner M.J., and Daughton S. (1990). Cerebral perfusion pressure management in head injury. J. Trauma 30, 933–940 2117669 10.1097/00005373-199008000-00001

[11] Robertson C., Valadka A.B., Hannay H.J., Contant C.F., Gopinath S.P., Cormio M., Uzura M., and Grossman R.G. (1999). Prevention of secondary ischemic insults after severe head injury. Crit. Care Med. 27, 2086–2095 10548187 10.1097/00003246-199910000-00002

[12] Naredi S., Olivecrona M., Lindgren C., Ostlund A.L., Grände P.O., and Koskinen L.O. (2001). An outcome study of severe traumatic head injury using the “Lund therapy” with low-dose prostacyclin. Acta Anaesthesiol. Scand. 45, 402–406 11300376 10.1034/j.1399-6576.2001.045004402.x

[13] Howells T., Elf K., Jones P.A., Ronne-Engström E., Piper I., Nilsson P., Andrews P., and Enblad P. (2005). Pressure reactivity as a guide in the treatment of cerebral perfusion pressure in patients with brain trauma. J. Neurosurg. 102, 311–317 10.3171/jns.2005.102.2.0311 15739560

[14] Czosnyka M., Smielewski P., Kirkpatrick P., Laing R.J., Menon D., and Pickard J.D. (1997). Continuous assessment of the cerebral vasomotor reactivity in head injury. Neurosurgery 41, 11–17 9218290 10.1097/00006123-199707000-00005

[15] Depreitere B., Güiza F., Van den Berghe G., Schuhmann M.U., Maier G., Piper I., and Meyfroidt G. (2014). Pressure autoregulation monitoring and cerebral perfusion pressure target recommendation in patients with severe traumatic brain injury based on minute-by-minute monitoring data. J. Neurosurg. 120, 1451–1457 24745709 10.3171/2014.3.JNS131500

[16] Steiner L.A., Czosnyka M., Piechnik K., Smielewski P., Chatfield D., Menon D.K., and Pickard J.D. (2002). Continuous monitoring of cerebrovascular pressure reactivity allows determination of optimal cerebral perfusion pressure in patients with traumatic brain injury. Crit. Care Med. 30, 733–738 11940737 10.1097/00003246-200204000-00002

[17] Zweifel C., Lavinio A., Steiner L.A., Radolovich D., Smielewski P., Timofeev I., Hiler M., Balestreri M., Kirkpatrick P.J., Pickard J.D., Hutchinson P., and Czosnyka M. (2008). Continuous monitoring of cerebrovascular pressure reactivity in patients with head injury. Neurosurg. Focus 25, E2 10.3171/FOC.2008.25.10.E2 18828700

[18] Aries M.J., Czosnyka M., Budohoski K.P., Steiner L.A., Lavinio A., Kolias A.G., Hutchinson P.J., Brady K.M., Menon D.K., Pickard J.D., and Smielewski P. (2012). Continuous determination of optimal cerebral perfusion pressure in traumatic brain injury. Crit. Care Med. 40, 2456–2463 22622398 10.1097/CCM.0b013e3182514eb6

[19] Piper I., Citerio G., Chambers I., Contant C., Enblad P., Fiddes H., Howells T., Kiening K., Nilsson P., and Yau Y.H. (2003). The Brain-IT group: concept and core dataset definition. Acta Neurochirurg. (Wien) 145, 615–628 10.1007/s00701-003-0066-6 14520540

[20] Jennett B., and Bond M. (1975). Assessment of outcome after severe brain damage. A practical scale. Lancet 1, 480–484 10.1016/s0140-6736(75)92830-5 46957

[21] Steyerberg E.W., Mushkudiani N., Perel P., Butcher I., Lu J., McHugh G.S., Murray G.D., Marmarou A., Roberts I., Habbema J.D., and Maas A.I. (2008). Predicting outcome after traumatic brain injury: development and international validation of prognostic scores based on admission characteristics. PLoS Med. 5, e165 18684008 10.1371/journal.pmed.0050165 PMC2494563

[22] Brain Trauma Foundation; American Association of Neurological Surgeons; Congress of Neurological Surgeons; Joint Section on Neurotrauma and Critical Care AANS/CNS, Bratton SL, Chestnut RM, Ghajar J, McConnell Hammond FF, Harris OA, Hartl R, Manley GT, Nemecek A, Newell DW, Rosenthal G, Schouten J, Shutter L, Timmons SD, Ullman JS, Videtta W, Wilberger JE, and Wright DW (2007). Guidelines for the management of severe traumatic brain injury. IX. Cerebral perfusion thresholds. J. Neurotrauma 24, S59–S64 17511547 10.1089/neu.2007.9987

[23] Drummond J.C. (1997). The lower limit of autoregulation: time to revise our thinking? Anesthesiology 86, 1431–1433 9197320 10.1097/00000542-199706000-00034

[24] Willie C.K., Tzeng Y.C., Fisher J.A., and Ainslie P.N. (2014). Integrative regulation of human brain blood flow. J. Physiol. 592, 841–859 24396059 10.1113/jphysiol.2013.268953 PMC3948549

[25] Tan C.O. (2012). Defining the characteristic relationship between arterial pressure and cerebral flow. J. Appl. Physiol. 113, 1194–1200 22961266 10.1152/japplphysiol.00783.2012 PMC3472492

[26] Numan T., Bain A.R., Hoiland R.L., Smirl J.D., Lewis N.C., and Ainslie P.N. (2014). Static autoregulation in humans: a review and reanalysis. Med. Eng. Phys. 36, 1487–1495 25205587 10.1016/j.medengphy.2014.08.001

[27] Tzeng Y.C., Willie C.K., Atkinson G., Lucas S.J., Wong A., and Ainslie P.N. (2010). Cerebrovascular regulation during transient hypotension and hypertension in humans. Hypertension 56, 268–273 20547971 10.1161/HYPERTENSIONAHA.110.152066

[28] Bouma G., Muizelaar J.P., Stringer W.A., Choi S.C., Fatouros P., and Young H.F. (1992). Ultra-early evaluation of regional cerebral blood flow in severely head-injured patients using xenon-enhanced computerized tomography. J. Neurosurg. 77, 360–368 1506882 10.3171/jns.1992.77.3.0360

[29] Sviri G.E., Aaslid R., Douville C.M., Moore A., and Newell D.W. (2009). Time course for autoregulation recovery following severe traumatic brain injury. J. Neurosurg. 111, 695–700 19392589 10.3171/2008.10.17686

