# Supplementary material for: Cerebral Perfusion Pressure Insults and Associations with Outcome in Adult Traumatic Brain Injury
Source: J Neurotrauma. 2017 Aug 15;34(16):2425–31. doi: 10.1089/neu.2016.4807 (PMC5563857; doi:10.1089/neu.2016.4807)
Supplement: Supplemental data [file Supp_Fig3.pdf]

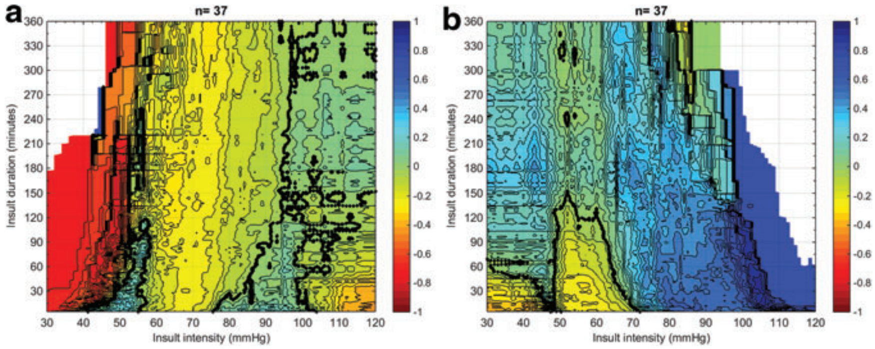

SUPPLEMENTARY FIG. S3. Visualization of correlation between Glasgow Outcome Score (GOS) and average number of cerebral perfusion pressure (CPP) insults for adults ≤65 years with decompressive craniectomy, $n = 37$. The univariate correlation between the average number of a certain CPP insult defined by severity (X-axis) and duration (Y-axis) and each GOS category is color-coded with blue representing a positive correlation and red representing a negative correlation. The contour of zero correlation is highlighted in black. 3a: insults of low CPP. 3b: insults of high CPP.
