# Supplementary material for: Cerebral Perfusion Pressure Insults and Associations with Outcome in Adult Traumatic Brain Injury
Source: J Neurotrauma. 2017 Aug 15;34(16):2425–31. doi: 10.1089/neu.2016.4807 (PMC5563857; doi:10.1089/neu.2016.4807)
Supplement: Supplemental data [file Supp_Fig4.pdf]

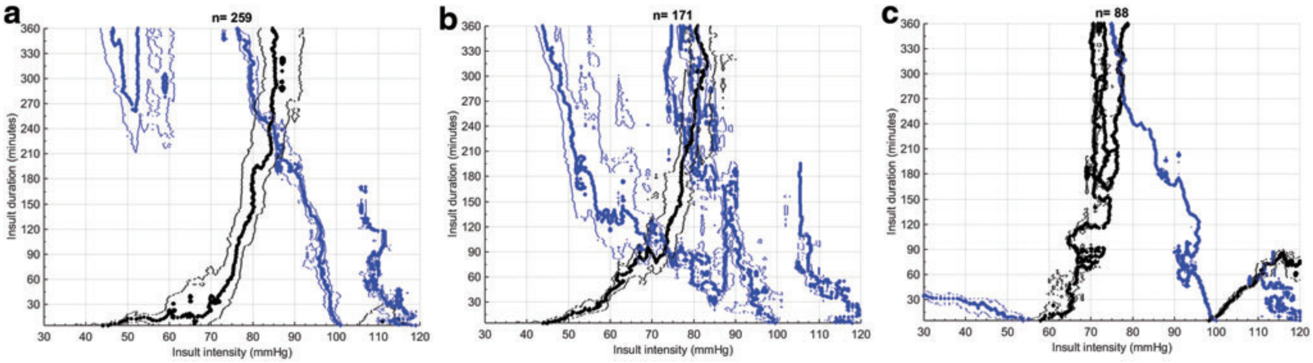

SUPPLEMENTARY FIG. S4. Comparison of cerebral perfusion pressure (CPP) insult transition curves according to centers' CPP distribution. Transition curves are the lines with 0 correlation between CPP insults and outcome. 4a: all centers ($n = 259$); 4b: centers with their median of patients' median CPP below 70 mm Hg ($n = 171$); 4c: centers with their median of patients' median CPP above 70 mm Hg ($n = 88$).
