# Supplementary material for: Cerebral Perfusion Pressure Insults and Associations with Outcome in Adult Traumatic Brain Injury
Source: J Neurotrauma. 2017 Aug 15;34(16):2425–31. doi: 10.1089/neu.2016.4807 (PMC5563857; doi:10.1089/neu.2016.4807)
Supplement: Supplemental data [file Supp_Table1.pdf]

Supplementary Table S1. Multivariate Model for Mortality Including the Cumulative Dose of Cerebral Perfusion Pressure Insult Burden Expressed as Percentage of Time Spent in the Red Zone ($n=259$)

	OR	*OR 95%*	*OR 95%*	p
Age	1.044	1.02	1.07	**0.0001**
GCS motor	0.86	0.70	1.07	0.17
Pupil reactivity	0.42	0.26	0.68	**0.0003**
% time in red zone	4.79	1.02	22.46	**0.04**

OR, odds ratio; GCS, Glasgow Coma Scale.

The percentage of monitoring time spent in the red zone was based on a new calculation, in which for each patient and for every minute of monitoring, it was checked whether this minute fulfilled criteria of belonging to one or more of the several episodes negatively associated with outcome (i.e. red zone), based on the transition curves from the graph for adults ≤65 years of age without decompressive craniectomy and using autoregulation active episodes only.
