# Supplementary material for: Cerebral Perfusion Pressure Insults and Associations with Outcome in Adult Traumatic Brain Injury
Source: J Neurotrauma. 2017 Aug 15;34(16):2425–31. doi: 10.1089/neu.2016.4807 (PMC5563857; doi:10.1089/neu.2016.4807)
Supplement: Supplemental data [file Supp_Fig5.pdf]

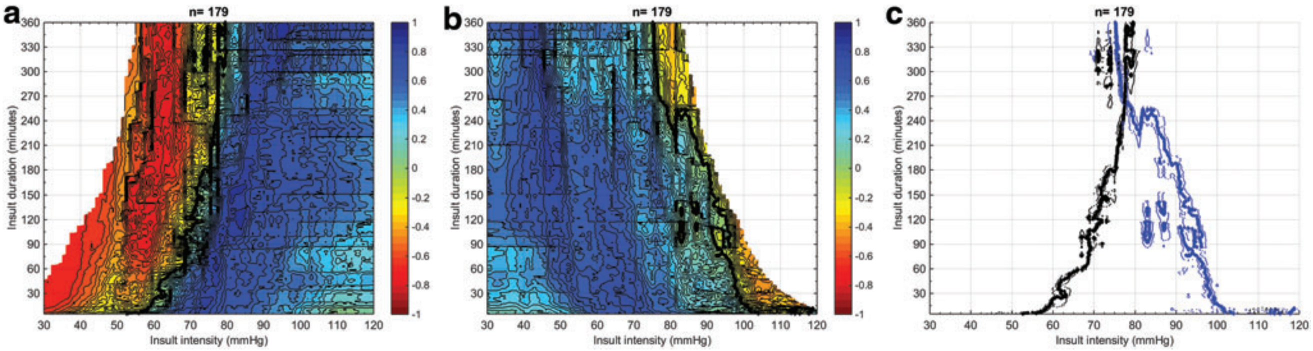

SUPPLEMENTARY FIG. S5. Visualization of correlation between Glasgow Outcome Score (GOS) and average number of cerebral perfusion pressure (CPP) insults for adults ≤65 years without decompressive craniectomy, $n=179$. The univariate correlation between the average number of a certain CPP insult defined by severity (X-axis) and duration (Y-axis) and each GOS category is color-coded with blue representing a positive correlation and red representing a negative correlation. The contour of zero correlation is highlighted in black. The plots were calculated correlating GOS with the daily averages of the number of insults instead of the absolute numbers of insults. 5a: insults of low CPP. 5b: insults of high CPP. 5c: low CPP (black) and high CPP (blue) transition curves plotted together.
